# The transcriptome response of the ruminal methanogen *Methanobrevibacter ruminantium* strain M1 to the inhibitor lauric acid

**DOI:** 10.1186/s13104-018-3242-8

**Published:** 2018-02-17

**Authors:** Xuan Zhou, Marc J. A. Stevens, Stefan Neuenschwander, Angela Schwarm, Michael Kreuzer, Anna Bratus-Neuenschwander, Johanna O. Zeitz

**Affiliations:** 1 Institute of Agricultural Sciences, ETH Zurich, Universitätstrasse 2, 8092 Zurich, Switzerland; 2 Laboratory of Food Biotechnology, Institute of Food, Nutrition and Health, ETH Zurich, Schmelzbergstrasse 7, 8092 Zurich, Switzerland; 3 Institute for Food Hygiene and Safety, University of Zurich, Winterthurerstrasse 272, 8057 Zurich, Switzerland; 4 Institute of Agricultural Sciences, ETH Zurich, Tannenstrasse 1, 8092 Zurich, Switzerland; 5 Functional Genomics Center Zurich, ETH Zurich and University of Zurich, Winterthurerstrasse 190, 8057 Zurich, Switzerland; 6 Institute of Animal Nutrition and Nutritional Physiology, Justus-Liebig University Giessen, 35392 Giessen, Germany

**Keywords:** *Methanobrevibacter ruminantium*, Methanogenesis, Fatty acid, Rumen, Gene expression, Lauric acid

## Abstract

**Objective:**

Lauric acid (C_12_) is a medium-chain fatty acid that inhibits growth and production of the greenhouse gas methane by rumen methanogens such as *Methanobrevibacter ruminantium*. To understand the inhibitory mechanism of C_12_, a transcriptome analysis was performed in *M. ruminantium* strain M1 (DSM 1093) using RNA-Seq.

**Results:**

Pure cell cultures in the exponential growth phase were treated with 0.4 mg/ml C_12_, dissolved in dimethyl sulfoxide (DMSO), for 1 h and transcriptomic changes were compared to DMSO-only treated cells (final DMSO concentration 0.2%). Exposure to C_12_ resulted in differential expression of 163 of the 2280 genes in the M1 genome (maximum log_2_-fold change 6.6). Remarkably, C_12_ hardly affected the expression of genes involved in methanogenesis. Instead, most affected genes encode cell-surface associated proteins (adhesion-like proteins, membrane-associated transporters and hydrogenases), and proteins involved in detoxification or DNA-repair processes. Enrichment analysis on the genes regulated in the C_12_-treated group showed a significant enrichment for categories ‘cell surface’ and ‘mobile elements’ (activated by C_12_), and for the categories ‘regulation’ and ‘protein fate’ (represssed). These results are useful to generate and test specific hypotheses on the mechanism how C_12_ affects rumen methanogens.

**Electronic supplementary material:**

The online version of this article (10.1186/s13104-018-3242-8) contains supplementary material, which is available to authorized users.

## Introduction

Ruminal methane-producing archaea acquire attention because ruminant livestock is estimated as the most important source of anthropogenic emission of the greenhouse gas methane [1]. Among the most-promising anti-methanogenic compounds are two medium chain fatty acids (MCFA), lauric acid (C_12_) and myristic acid (C_14_), which were shown to inhibit methanogenesis in vivo when supplemented to the diet of ruminants [2–4], in vitro in rumen fluid [5] and in methanogenic cultures [6]. MCFA cause leakage of K^+^ ions and decrease survival of *Methanobrevibacter ruminantium*, a dominant methanogen species in the rumen [6, 7]. Further, MCFA killed some, but not all methanogen cells, which implies that the cells may be capable to react to fatty acid-caused stress. In search of the mode of action, we investigated the transcriptional response of *M. ruminantium* to exposure of C_12_ in culture.

## Main text

### Methods

#### Experimental design

*Methanobrevibacter ruminantium* (strain M1, DSM 1093; ‘Deutsche Sammlung von Mikroorganismen und Zellkulturen’ (DSMZ), Braunschweig, Germany) was cultivated anaerobically in 50 ml of modified *Methanobacterium* medium (DSMZ No. 1523) in 116 ml bottles under a CO_2_/H_2_ (0.2:0.8) atmosphere at 150 kPa and at 39 °C in an incubation shaker as described previously [6]. Growth of the cultures was monitored by recording optical density at 600 nm and by methane (CH_4_) formation after 24, 48, 60 and 61 h. The culture was inoculated with 5 ml of an exponentially growing pre-culture (OD_600_ ~ 0.64) to 45 ml of medium. Cell survival was detected with the LIVE/DEAD BacLight Bacterial Viability Kit for microscopy and quantitative assays (Kit L7012; Invitrogen GmbH, Darmstadt, Germany) [6]. Lauric acid (≥ 97% purity) was obtained from Sigma-Aldrich (Buchs, Switzerland), and a stock solution with 200 mg/ml was prepared by dissolving the C_12_ in sterile dimethyl sulfoxide (DMSO) (Sigma-Aldrich), a commonly used solvent for water-insoluble substances [8]. After 60 h of incubation, when cells reached the exponential phase, three bottles were supplemented with 0.1 ml of the C_12_ stock solution to reach a final concentration of 0.4 mg C_12_/ml (treatment group), three bottles were supplemented with 0.1 ml of DMSO (final concentration: 0.2%) (control group), and three bottles received no supplement (blank group). The concentration of C_12_ and the exposure time of 1 h chosen were in a range where most cells remained alive and where CH_4_ formation was clearly but not completely inhibited. It was verified that, at 61 h of incubation, CH_4_ formation rates and proportion of living cells did not differ between DMSO-exposed control cultures (measured: 0.71 ± 0.03 µmol/ml × h and 97 ± 0.3%, respectively) and untreated blank cultures (0.74 ± 0.04 µmol/ml × h and 99 ± 1.2%). At 61 h, i.e. after 1 h of exposure to C_12_, CH_4_ formation rates in the hour after exposure were suppressed by 40 ± 6% compared to the control cultures (*P* < 0.05), and cell viability was reduced down to 71 ± 1.8% when compared to the control cultures (P < 0.05). At this time point, three samples per group (each 50 ml of culture) were anaerobically collected at 4 °C after centrifugation at 5000×*g* for 6 min. Cell pellets were immediately frozen in liquid nitrogen and stored at − 80 °C until RNA extraction.

#### RNA isolation

Total RNA was isolated from the frozen cell pellets by using TRIzol^®^ Reagent (ThermoFisher, Waltham, MS, USA), according to the manufacturer’s protocol. In order to remove genomic DNA from total RNA samples, a DNA digestion was performed with the RNase-Free DNase Set (Qiagen, Hilden, Germany) following manufacturer’s instructions. Quantity and quality of extracted RNA were determined by a Qubit^®^ 1.0 fluorometer with a Qubit RNA BR (Broad Range) assay kit (Invitrogen, Carlsbad, CA, USA) and by an Agilent 2200 TapeStation with the Agilent RNA ScreenTape assay (Agilent Technologies, Santa Clara, CA, USA), respectively. Nine purified total RNA samples with a yield of at least 5 µg and RNA integrity numbers (RIN) in a range of 5.6–7.6 were used for sequencing. These included three replicates per group: three DMSO-dissolved C_12_-treated samples (T1, T2 and T3), three samples with DMSO supplementation alone (control samples C1, C2, C3) and three samples without supplement (blank samples B1, B2, B3).

#### Ribosomal RNA depletion

The Ribo-Zero™ rRNA removal kit (Bacteria) (http://www.illumina.com/products/ribo-zero-rrna-removal-bacteria.html, Epicentre, San Diego, USA) was applied to deplete rRNA from the *M. ruminantium* total RNA samples (5 µg) by following the Illumina user guide for the Ribo-Zero Magnetic kits (Part#15065382 Rev. A, November 2014). The rRNA-depleted samples were purified with AMPure RNAClean XP Beads (Beckman-Coulter Genomics, Nyon, Switzerland) as recommended in the Illumina protocol mentioned above.

#### Next generation sequencing

Enriched RNA samples were used to produce library constructs by following the Illumina TruSeq^®^ Stranded total RNA protocol (Part#15031048 Rev. C, September 2012) with the Illumina TruSeq Stranded total RNA Sample Preparation Kit. Libraries were quantified and quality checked using qPCR with Illumina adapter specific primers (Roche LightCycler^®^ system, Roche Diagnostics, Basel, Switzerland) and by the Agilent Technologies 2100 Bioanalyzer with DNA-specific chips, respectively. Diluted indexed libraries (10 nM) were pooled, used for cluster generation (Illumina TruSeq SR Cluster Kit v4-cBot-HS reagents) and further sequenced (Illumina TruSeq SBS Kit v4-HS reagents) on the Illumina HiSeq 2500 instrument in the high output mode according to the manufacturer’s recommendations. Illumina single read approach (1 × 125 bp) was used to generate raw sequencing reads with a depth of approximately 20–30 million reads per sample.

#### RNA-sequencing data analysis

Data analyses were performed as described by Tanner et al. [9]. Shortly, reads (125 bp) were mapped against the genome of *M. ruminantium* M1 using the CLC Genomics Workbench 6.5.1 (CLC, Aarhus, Denmark). Statistical analysis was performed using Bioconductor EdgeR software package in R. A false discovery rate (FDR) value < 0.05 was used as cutoff for significance of differentially expressed genes and log_2_ fold change > 1 and < −1 was used as cutoff for differential transcription of genes higher (positive log_2_-fold change values) or lower (negative log_2_-fold change values) expressed in cultures [10]. To test for significant enrichment in each category listed in Table 1, a two-tailed Fisher test was performed at http://www.langsrud.com/fisher.htm.

**Table 1:** Number of genes significantly differential expressed within functional categories

Category	Gene count	Treatment vs. control		Control vs. blank		Treatment vs. blank	
		Up	Down	Up	Down	Up	Down
Amino acid metabolism	94	2^b^	3	0	4	1	2
Cell cycle	29	1	0	0	0	0	0
Cell envelope	189	28^a^	0^b^	2	4	2	3
Cellular processes	14	3	1	1	0	2^a^	0
Central carbon metabolism	61	2	1	0	1	2	0
Energy metabolism	141	9	9^a^	6	3	6	0
Lipid metabolism	21	0	0	1	0	0	3^a^
Mobile elements	87	37^a^	0	0	37^a^	0	0
Nitrogen metabolism	14	0	1	1	0	1	0
Nucleic acid metabolism	60	2	1	0	0	0	0
Protein fate	51	0^b^	2	1	0	1	0
Protein synthesis	169	7	1	0^b^	9	0^b^	0
Purines and pyrimidines	47	2	0	0	0	0	0
Regulation	68	0^b^	5^a^	5^a^	0	2	0
Secondary metabolites	12	4	0	0	0	0	0
Transcription	26	1	0	0	0	0	0
Transporters	97	11	1	7^a^	3	7^a^	1
Unknown function	183	10	8	4	2^b^	3	0
Vitamins and cofactors	142	8	3	2	4	5	1
Total^c^	1505	127	36	30	67	32	10

^a^Significant functional enrichment in a Fisher exact test (p < 0.05)

^b^Significant functional underrepresentation in a Fisher exact test (p < 0.05)

^c^Non-conserved hypothetical genes and RNAs are omitted in the classification [11]. Treatment: with DMSO-dissolved C_12_, control: with DMSO alone, blank: without C_12_ and DMSO

### Results and discussion

**Fig. 1:**
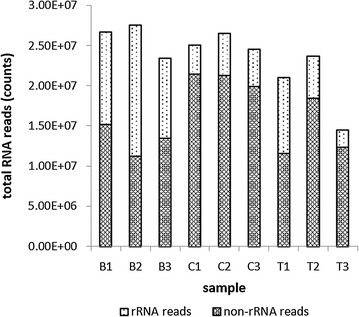
Ribosomal RNA depletion and reads enrichment in RNA extracted from *M. ruminantium* M1. B: blank (without C_12_ and dimethyl sulfoxide, DMSO), C: control (with DMSO alone), T: treatment (with DMSO-dissolved C_12_). Note that the y-axis is non-linear

The Ribo-Zero™ rRNA Removal Kit can be used to efficiently remove the rRNA fraction from total RNA samples isolated from the archaeon *M. ruminantium* M1. The Epicentre probes (directed to bind rRNA from a broad spectrum of bacteria species) reduced the rRNAs in all samples tested, which resulted in 40–85% of non-rRNA sequencing reads in the samples (Fig. 1). More than 10 million mRNA sequencing reads per sample were mapped to the genome of *M. ruminantium* M1 (Fig. 1), which is a sufficient coverage for transcriptome analyses [11].

First, we compared the untreated cultures to the control cultures treated with DMSO. DMSO affected the expression of 97 out of 2280 genes in the M1 genome (Additional file 1). DMSO induced changes in gene expression of cell surface-related proteins, cell membrane-associated transporters and intracellular proteins; the latter maybe related to the observation that DMSO penetrates cell membranes [8]. DMSO-regulated genes included genes encoding proteins related to the cell envelope, mainly adhesion-like proteins (six genes; four down-regulated, two up-regulated). Others were classified as mobile genetic elements (38 genes including hypothetical genes; all down-regulated), and genes involved in energy metabolism, mainly hydrogen metabolism [nine genes, six up-regulated (frhA/B1/D/G, mtrA2, DsbD), three down-regulated (hypA/B, adh3)]. Genes involved in metabolism of vitamins and cofactors (six genes; four down-regulated, two up-regulated) as well as of amino acids (four genes, all down-regulated) were regulated. Moreover, cation transporters (five genes; four of five up-regulated), amino acid transporters (two genes; down-regulated), and other transporters (three genes, up-regulated) showed differential expression when untreated cultures were compared to DMSO-supplemented cultures. Overall, the set of genes regulated in the DMSO control group compared to the blank group was enriched for genes assigned to categories: ‘Mobile elements’, ‘Transporters’, and ‘Regulation’, whereas genes assigned to ‘protein synthesis’ and genes of unknown function were significantly underrepresented (Table 1).

The comparison between the C_12_ + DMSO-treated and the untreated cultures revealed 42 genes differentially regulated (Additional file 2), 26 of these also found in the DMSO-treated versus untreated comparison (Additional file 3).

Thereafter the transcriptome of the C_12_ + DMSO-treated and DMSO-treated cultures were compared to identify the mechanisms how MCFA affect methanogenesis. A total of 147 genes, 6.4% of all 2280 genes, were differentially regulated (Table 2).

**Table 2:** Significant changes of gene expression in *M. ruminantium* M1 cultures exposed to C_12_

Category and subcategory	ORF	Gene name	Annotated function	log2-fold change	log2 counts per 106 reads
Amino acid metabolism					
Lysine	mru_0152	lysA	Diaminopimelate decarboxylase LysA	− 1.02	7.66
	mru_0153	dapF	Diaminopimelate epimerase DapF	− 1.00	7.01
Histidine	mru_0182	hisH	Imidazole glycerol phosphate synthase glutamine amidotransferase subunit HisH	− 1.07	6.27
Serine	mru_0678	serA	Phosphoglycerate dehydrogenase SerA	1.03	9.59
Tryptophan	mru_2159	trpB2	Tryptophan synthase beta subunit TrpB2	1.00	11.31
Cell cycle					
Cell division	mru_2160	minD	Cell division ATPase MinD	1.08	5.46
Cell envelope					
Cell surface proteins	mru_1500	mru_1500	Adhesin-like protein	1.00	8.58
	mru_0160	mru_0160	Adhesin-like protein	1.02	6.70
	mru_0963	mru_0963	Adhesin-like protein	1.08	12.13
	mru_1263	mru_1263	Adhesin-like protein	1.15	9.15
	mru_0331	mru_0331	Adhesin-like protein	1.15	10.34
	mru_0338	mru_0338	Adhesin-like protein	1.17	8.55
	mru_1124	mru_1124	Adhesin-like protein	1.20	12.55
	mru_0031	mru_0031	Adhesin-like protein	1.27	11.29
	mru_0687	mru_0687	Adhesin-like protein	1.28	10.46
	mru_0245	mru_0245	Adhesin-like protein	1.32	8.78
	mru_1417	mru_1417	Adhesin-like protein	1.43	9.49
	mru_1650	mru_1650	Adhesin-like protein	1.44	4.24
	mru_1465	mru_1465	Adhesin-like protein	1.61	6.82
	mru_1506	mru_1506	Adhesin-like protein	1.61	7.76
	mru_0417	mru_0417	Adhesin-like protein	1.70	5.86
	mru_0327	mru_0327	Adhesin-like protein	1.73	10.86
	mru_0019	mru_0019	Adhesin-like protein	2.04	7.42
	mru_0084	mru_0084	Adhesin-like protein	2.07	6.71
	mru_2049	mru_2049	Adhesin-like protein	2.25	11.23
	mru_2043	mru_2043	Adhesin-like protein	2.27	8.58
	mru_1726	mru_1726	Adhesin-like protein	2.32	8.37
	mru_2090	mru_2090	Adhesin-like protein	2.51	13.88
	mru_2147	mru_2147	Adhesin-like protein	2.73	13.13
	mru_0326	mru_0326	Adhesin-like protein	5.04	12.58
	mru_0015	mru_0015	Adhesin-like protein with cysteine protease domain	1.49	9.07
	mru_0020	mru_0020	Adhesin-like protein with cysteine protease domain	2.78	7.86
Teichoic acid biosynthesis	mru_1079	mru_1079	CDP-glycerol:poly(glycerophosphate) glycerophosphotransferase	1.27	6.32
Pseudomurein biosynthesis	mru_1118	mru_1118	Cell wall biosynthesis protein Mur ligase family	1.07	9.37
Cellular processes					
Oxidative stress response	mru_1507	fprA1	F420H2 oxidase FprA1	1.37	10.47
	mru_0131	fprA2	F420H2 oxidase FprA2	3.58	12.42
	mru_1367	rbr2	Rubrerythrin Rbr2	1.27	13.19
Stress response	mru_0183	mru_0183	Protein disulfide-isomerase thioredoxin-related protein	− 1.19	7.79
Central carbon metabolism					
Gluconeogenesis	mru_0628	pgk2A	2-Phosphoglycerate kinase Pgk2A	1.85	7.69
Other	mru_1685	deoC	Deoxyribose-phosphate aldolase DeoC	5.12	11.11
Acetate	mru_1786	mru_1786	Transporter SSS family	− 1.18	8.66
Energy metabolism					
Electron transfer	mru_0915	mru_0915	4Fe–4S binding domain-containing protein	− 1.06	7.64
	mru_2036	mru_2036	4Fe–4S binding domain-containing protein	1.25	5.60
	mru_1345	mru_1345	4Fe–4S binding domain-containing protein	1.30	7.63
Methanogenesis pathway	mru_0569	mer	5,10-methylenetetrahydro-methanopterin reductase Mer	− 1.36	12.71
	mru_0526	hmd	Coenzyme F420-dependent *N*(5), *N*(10)-methenyltetrahydromethanopterin reductase Hmd	1.41	10.96
	mru_1850	atwA2	Methyl-coenzyme M reductase component A2 AtwA2	1.05	10.86
	mru_1927	mcrD	Methyl-coenzyme M reductase D subunit McrD	− 1.43	11.33
	mru_0441	mtrA2	Tetrahydromethanopterin *S*-methyltransferase subunit A MtrA2	− 2.14	11.99
	mru_1918	mtrF	Tetrahydromethanopterin *S*-methyltransferase subunit F MtrF	− 1.24	9.71
Electron transfer	mru_0184	dsbD	Cytochrome C-type biogenesis protein DsbD	− 1.16	6.17
	mru_0830	mru_0830	Ferredoxin	2.56	9.31
H2 metabolism	mru_1410	ehaC	Energy-converting hydrogenase A subunit C EhaC	− 1.63	6.30
	mru_1408	ehaE	Energy-converting hydrogenase A subunit E EhaE	− 1.74	7.34
	mru_1632	hypB	Hydrogenase accessory protein HypB	2.25	7.90
	mru_1633	hypA	Hydrogenase nickel insertion protein HypA	2.19	7.47
Formate metabolism	mru_0332	fdhC	Formate/nitrite transporter FdhC	− 1.11	11.98
Alcohol metabolism	mru_1445	adh3	NADP-dependent alcohol dehydrogenase Adh3	6.42	7.81
	mru_1444	npdG2	NADPH-dependent F420 reductase NpdG2	3.84	5.32
Mobile elements					
Prophage	mru_0269	mru_0269	ATPase involved in DNA replication control MCM family	2.51	4.60
	mru_0323	mru_0323	dnd system-associated protein 2	1.11	6.63
	mru_0280	mru_0280	ParB-like nuclease domain-containing protein	2.52	1.87
	mru_0256	mru_0256	Phage integrase	1.69	6.95
	mru_0287	mru_0287	Phage portal protein	2.73	1.86
	mru_0315	mru_0315	Phage tail tape measure protein	2.47	3.39
	mru_0270	mru_0270	Phage-related protein	1.91	4.54
	mru_0288	mru_0288	Phage-related protein	2.21	2.32
	mru_0058	mru_0058	Phage-related protein	2.53	− 0.04
	mru_0282	mru_0282	Phage-related protein	2.64	1.93
	mru_0316	mru_0316	Phage-related protein	2.66	3.40
	mru_0317	mru_0317	Phage-related protein	2.89	3.42
	mru_0311	mru_0311	Phage-related protein	3.14	2.55
	mru_0310	mru_0310	Phage-related protein	3.18	1.56
	mru_0284	mru_0284	Phage-related protein	3.35	1.93
	mru_0307	mru_0307	Phage-related protein	3.38	2.86
	mru_0313	mru_0313	Phage-related protein	3.40	2.83
	mru_0308	mru_0308	Phage-related protein	3.48	3.46
	mru_0324	mru_0324	Type II restriction enzyme, methylase subunit	1.88	5.99
CRISPR-associated genes	mru_0798	mru_0798	CRISPR-associated protein Cas1-1	1.93	4.09
	mru_1181	mru_1181	CRISPR-associated RAMP protein Csm3 family	1.03	7.23
Nitrogen metabolism					
Other	mru_2121	hcp	Hydroxylamine reductase Hcp	− 1.46	12.26
Nucleic acid metabolism					
Helicase	mru_0981	mru_0981	Rad3-related DNA helicase	1.09	7.97
Recombination and repair	mru_2097	recJ1	ssDNA exonuclease RecJ1	1.39	11.06
	mru_1383	mru_1383	Staphylococcal nuclease domain-containing protein	− 1.30	7.06
Protein fate					
Protein folding	mru_1511	mru_1511	Nascent polypeptide-associated complex protein	− 1.00	6.61
Protein secretion	mru_1581	mru_1581	Signal peptidase I	− 1.21	7.34
Protein synthesis					
RNA processing	mru_0589	mru_0589	NMD3 family protein	1.50	7.52
Translation factors	mru_0728	mru_0728	Peptide chain release factor aRF1	1.46	7.74
Ribosomal proteins	mru_0865	rpl5p	Ribosomal protein L5P Rpl5p	1.03	8.24
	mru_0868	rpl6p	Ribosomal protein L6P Rpl6p	1.05	7.92
	mru_2098	mru_2098	Ribosomal protein S15P Rps15p	1.19	9.21
Other	mru_0519	mru_0519	RNA-binding protein	− 1.68	8.08
	mru_1978	mru_1978	RNA-metabolising metallo-beta-lactamase	1.58	8.74
RNA processing	mru_1846	dusA2	tRNA-dihydrouridine synthase DusA2	1.06	6.58
Purines and pyrimidines					
Interconversion	mru_2104	surE1	5′-Nucleotidase SurE1	1.02	7.02
	mru_0241	nrdD	Anaerobic ribonucleoside-triphosphate reductase NrdD	1.47	11.08
Regulation					
Protein interaction	mru_1186	mru_1186	TPR repeat-containing protein	− 1.05	8.81
Transcriptional regulator	mru_2122	mru_2122	Transcriptional regulator	− 1.62	8.68
	mru_1447	mru_1447	Transcriptional regulator	− 1.55	8.56
	mru_1446	mru_1446	Transcriptional regulator ArsR family	− 1.21	7.78
	mru_0442	mru_0442	Transcriptional regulator MarR family	− 1.68	4.74
Secondary metabolites					
Other	mru_0514	mru_0514	4′-Phosphopantetheinyl transferase family protein	1.26	6.32
	mru_0069	mru_0069	MatE efflux family protein	1.20	7.17
	mru_0352	mru_0352	MatE efflux family protein	1.64	6.73
NRPS	mru_0351	mru_0351	Non-ribosomal peptide synthetase	1.06	10.17
Transcription					
RNA polymerase	mru_0161	rpoF	DNA-directed RNA polymerase subunit F RpoF	1.05	9.66
Transporters					
Amino acids	mru_1775	mru_1775	Amino acid ABC transporter ATP-binding protein	1.03	5.46
	mru_1776	mru_1776	Amino acid ABC transporter permease protein	1.25	4.94
Cations	mru_1861	mru_1861	Heavy metal translocating P-type ATPase	− 6.61	10.24
	mru_1706	nikD2	Nickel ABC transporter ATP-binding protein NikD2	1.15	6.54
	mru_1617	nikB1	Nickel ABC transporter permease protein NikB1	1.10	7.35
	mru_1709	nikB2	Nickel ABC transporter permease protein NikB2	1.43	7.34
	mru_1708	nikC2	Nickel ABC transporter permease protein NikC2	1.31	7.03
	mru_1710	nikA2	Nickel ABC transporter substrate-binding protein NikA2	1.14	11.86
Other	mru_0253	mru_0253	ABC transporter ATP-binding protein	1.97	7.23
	mru_0252	mru_0252	ABC transporter permease protein	1.71	7.40
	mru_0251	mru_0251	ABC transporter substrate-binding protein	2.06	9.13
	mru_0329	mru_0329	MotA/TolQ/ExbB proton channel family protein	1.56	6.00
Vitamins and cofactors					
Biotin	mru_0527	bioB2	Biotin synthase BioB2	1.24	7.09
Cobalamin	mru_0539	cbiM1	Cobalamin biosynthesis protein CbiM1	1.21	9.82
	mru_0540	cbiN1	Cobalt transport protein CbiN1	1.18	8.30
	mru_0360	cbiA1	Cobyrinic acid a,c-diamide synthase CbiA1	− 1.60	8.09
	mru_1852	cysG	Siroheme synthase CysG	1.20	7.47
Coenzyme B	mru_0385	aksA	Homocitrate synthase AksA	− 1.15	10.22
Metal-binding pterin	mru_0200	modB	Molybdate ABC transporter permease protein ModB	2.04	9.37
	mru_0201	modA	Molybdate ABC transporter substrate-binding protein ModA	2.83	10.54
Thiamine	mru_0247	thiC1	Thiamine biosynthesis protein ThiC1	− 1.18	9.24
	mru_0532	mru_0532	ThiF family protein	1.38	4.67
Others	mru_1769	nifB	Nitrogenase cofactor biosynthesis protein NifB	2.58	8.89
Unknown function					
Enzyme	mru_0455	mru_0455	Acetyltransferase	− 1.16	9.80
	mru_1758	mru_1758	Acetyltransferase	− 1.10	6.05
	mru_2170	mru_2170	Acetyltransferase	1.32	6.12
	mru_0574	mru_0574	Acetyltransferase GNAT family	− 1.92	1.81
	mru_1707	mru_1707	Acetyltransferase GNAT family	1.48	5.54
	mru_0560	mru_0560	ATPase	1.11	8.14
	mru_1613	mru_1613	SAM-dependent methyltransferase	1.58	4.18
Other	mru_0231	mru_0231	CAAX amino terminal protease family protein	− 1.09	8.53
	mru_1993	mru_1993	CBS domain-containing protein	− 1.65	10.72
	mru_1994	mru_1994	CBS domain-containing protein	− 1.31	11.57
	mru_0474	mru_0474	HD domain-containing protein	1.33	7.47
	mru_1034	mru_1034	HEAT repeat-containing protein	2.35	8.75
	mru_2109	mru_2109	Methanogenesis marker protein 12	− 1.01	7.90
	mru_0562	mru_0562	PP-loop family protein	1.59	7.50
	mru_1678	mru_1678	Redox-active disulfide protein	1.51	7.12
	mru_0561	mru_0561	Von Willebrand factor type A domain-containing protein	1.33	8.52
	mru_1510	mru_1510	YhgE/Pip-like protein	− 1.31	8.45
	mru_0627	mru_0627	ZPR1 zinc-finger domain-containing protein	2.04	6.70

C_12_-treated cultures were compared to DSMO-exposed control cultures (significant change with log_2_fold changes < 1 and > 1 and a false discovery rate < 0.05). The list does not include the 71 regulated hypothetical proteins. The *M. ruminantium* (mru) open reading frame (ORF) codes are adopted from the Kyoto Encyclopedia of Genes and Genomes

The subcellular localization of the encoded protein could be identified for 75% of the regulated genes. Predominantly, genes associated with the cell envelope were affected, namely trans-membrane proteins or membrane-associated proteins. Enrichment analysis showed that, with C_12_ exposure, mainly adhesion-like proteins (category ‘cell surface’) and phage-related proteins (‘mobile elements’) were significantly enriched in the regulated genes data set (Table 1). This supports earlier suggestions that MCFA primarily target the cell envelope and processes that occur at the cell membrane [12]. For example, upon exposure to C_12_ in the present study, the mRNA abundance of 26 adhesion-like proteins (ALPs) (part of the cell envelope [13]), i.e. of 25% of all ALPs of *M. ruminantium*, and of two proteins involved in biosynthesis of teichoic acid and pseudomurein which are cell-wall related [14], were up-regulated compared to the DMSO control group (Table 2).

Two subunits of the membrane-bound energy-converting hydrogenase (Eha), which is involved in hydrogenotrophic methanogenesis [13, 15], were down-regulated by log_2_1.6- and 1.7-fold in cultures exposed to C_12_, whereas two cytoplasmic hydrogenases (Frh, Mvh) were not. A gene encoding ferredoxin, a trans-membrane iron-sulfur protein involved in electron transfer from hydrogen, was up-regulated (log 2.6-fold upon C_12_ exposure). Expression of 3 genes encoding trans-membrane 4Fe-4S binding domain-containing proteins was affected by C_12_ exposure. Two subunits of the methyl-H4MPT:coenzyme M methyltransferase (Mtr), which is membrane-bound and plays a crucial role in the methanogenesis pathway [15, 16], were down-regulated by log_2_ 2.1- and 1.2-fold upon C_12_ exposure. In total 13 genes encoding mainly transporters of amino acids and cations displayed differences in transcript abundance after C_12_ exposure (Table 2). For example, several genes encoding subunits of cations transporters, like the nickel ABC transporter permease proteins or nickel ABC transporter ATP-binding proteins, NikA2, NikB1, NikB2, NikC2 and NikD2, were differentially regulated. These cation transporters belong to a large family of ABC transporters (peptide/nickel transporter family) in ABC-type nickel transporter system, which is composed of a periplasmic binding protein (NikA), two integral membrane proteins (NikB and NikC) and two ABC proteins (NikD and NikE) [17]. One P-type ATPase, which are membrane-bound efflux pumps involved in metal homeostasis of microorganisms [18], was down-regulated. In prokaryotes, ABC transporters and P-type ATPases have important functions in maintaining appropriate concentrations of transition metals such as Ni, Co, Fe, Cu, and Zn, which are essential components of many prokaryotic enzymes [18]. Two transmembrane cobalt transport proteins (mru_0540; mru_0539), and two membrane-associated proteins involved in molybdate transport (mru_0200, mru_0201) [19], were up-regulated.

In addition, genes encoding intracellular proteins were affected by C_12_ exposure. These data support earlier observations that exposure to C_12_ causes leakage of intracellular K^+^ ions in *M. ruminantium* [6, 7], thus damages the cell envelope. Amongst the regulated genes, mostly genes encoding proteins involved in DNA repair, and genes controlling transcription/translation and redox homeostasis were affected. For example, thioredoxins and rubrerythrins showed an altered expression; they are considered to form a system protecting Archaea against oxidative stress [20, 21]. Thioredoxin-like proteins exhibit biochemical activities similar to thioredoxin and help methanogens maintain redox homeostasis [7]. Genes which were up-regulated by C_12_ included genes encoding proteins that are involved in nucleic acid metabolism and repair and in translation include a helicase (mru_0981), an exonuclease (mru_2097, recJ1), an anaerobic ribonucleosid-triphosphate reductase nrdD (mru_0241), a nucleotidase (mru_2104; SurE1), and a RNA-metabolizing metallo-beta-lactamase (mru_1978). Several genes involved in translation or post-translational modification were down-regulated, e.g. a staphylococcal nuclease domain-containing protein (mru_1383), a nascent polypeptide-associated complex protein (mru_1511), an RNA-binding protein (mru_0519) and a signal peptidase (mru_1581).

## Conclusion

The transcriptional response of *M. ruminantium* to the fatty acid C_12_ does not involve repression of specific pathway such as the methanogenesis pathway. Instead, it implies that C_12_ provokes broad transcriptional changes, and targets primarily cell surface associated adhesion-like proteins, phage-related proteins, and transmembrane proteins. How this response affects methanogens remains unclear. Future studies may investigate how different dosages of and prolonged exposure to C_12_ affect gene and protein expression and survival of *M. ruminantium*.

### Limitations

One limitation of our study is the low number of replicates per group. In addition, only one dosage of C_12_ was tested and samples for RNA sequencing were collected only at one time point; this precludes generalization to situations where C_12_ affects *M. ruminantium* stronger or weaker.

## Additional files

**Additional file 1: Table S1.** *M. ruminantium* M1 genes with significantly changed expression of genes in the DMSO control as compared to the blank group (log_2_-fold change < 1 and > 1, false discovery rate < 0.05). The list does not include the 59 regulated hypothetical proteins. The *M. ruminantium* (mru) open reading frame (ORF) codes are adopted from the Kyoto Encyclopedia of Genes and Genomes.

**Additional file 2: Table S2.** *M. ruminantium* M1 genes with significantly changed expression of genes in the cultures exposed to C_12_ + DMSO as compared to the blank group (log_2_-fold change < 1 and > 1, false discovery rate < 0.05). The list does not include the 15 regulated hypothetical proteins. The *M. ruminantium* (mru) open reading frame (ORF) codes are adopted from the Kyoto Encyclopedia of Genes and Genomes.

**Additional file 3: Figure S1.** Venn diagram indicates the number of differentially expressed genes between the experimental groups and the common overlapping differentially expressed genes. TC: treatment (C_12_ + DMSO) vs. control (DMSO); TB: treatment (C_12_ + DMSO) vs. untreated blank; CB: control (DMSO) vs. untreated blank. It should be kept in mind that it is not possible to distinguish between the DMSO and the C_12_ effect in the dataset comparing the treatment and the blank samples, and that the C_12_ effect is much better studied in the TC comparison (C_12_ + DMSO vs DMSO). The DMSO effect can be partial quenched by the C_12_ effect, so genes regulated in CB and TC are not necessarily regulated in the TB. The 26 common genes differentially expressed in *M. ruminantium* exposed to DMSO or DMSO + C_12_ compared to the untreated blank control are outlined in the tables on the right side. The 35 overlapping differentially expressed genes of the TC and CB comparisons are outlined in the table on the left side. The diagram was generated using the online tool at bioinformatics.psb.ugent.be/webtools/Venn/.

## Abbreviations

MCFA: medium-chain fatty acids
C_12_: lauric acid
DMSO: dimethyl sulfoxide
CH_4_: methane
