# Supplementary material for: The transcriptome response of the ruminal methanogen Methanobrevibacter ruminantium strain M1 to the inhibitor lauric acid
Source: BMC Res Notes. 2018 Feb 17;11:135. doi: 10.1186/s13104-018-3242-8 (PMC5816558; doi:10.1186/s13104-018-3242-8)
Supplement: Supplementary file 1 — Additional file 1: Table S1. M. ruminantium M1 genes with significantly changed expression of genes in the DMSO control as compared to the blank group (log2-fold change < 1 and > 1, false discovery rate < 0.05). The list does not include the 59 regulated hypothetical proteins. The M. ruminantium (mru) open reading frame (ORF) codes are adopted from the Kyoto Encyclopedia of Genes and Genomes. [file 13104_2018_3242_MOESM1_ESM.docx]

**Additional file 1: Table S1.** ***M. ruminantium* M1 genes with significantly changed expression of genes in the DMSO control as compared to the blank group (log_2_-fold change < 1 and > 1, false discovery rate < 0.05).** The list does not include the 59 regulated hypothetical proteins. The *M. ruminantium* (mru) open reading frame (ORF) codes are adopted from the Kyoto Encyclopedia of Genes and Genomes.

| **CATEGORY and Subcategory** | **ORF** | **Gene name** | **Annotated function** | **log2-fold change** | **log2 counts per 10^6^ reads** |
| --- | --- | --- | --- | --- | --- |
| AMINO ACID METABOLISM | |  |  |  |  |
| Methionine | mru_1569 | mru_1569 | O-acetylhomoserine/O-acetylserine sulfhydrylase MetZ/CysK2 | -1.35 | 6.79 |
| Tryptophan | mru_0212 | trpF | phosphoribosylanthranilate isomerase TrpF | -1.26 | 6.98 |
|  | mru_0214 | trpA | tryptophan synthase alpha subunit TrpA | -1.41 | 8.71 |
|  | mru_0213 | trpB1 | tryptophan synthase beta subunit TrpB1 | -1.20 | 8.82 |
| CELL ENVELOPE |  |  |  |  |  |
| Cell surface proteins | mru_2147 | mru_2147 | adhesin-like protein | -1.10 | 11.97 |
|  | mru_1124 | mru_1124 | adhesin-like protein | -1.08 | 12.57 |
|  | mru_2049 | mru_2049 | adhesin-like protein | -1.03 | 10.42 |
|  | mru_0327 | mru_0327 | adhesin-like protein | 1.35 | 9.34 |
|  | mru_0245 | mru_0245 | adhesin-like protein | 1.44 | 7.55 |
| Expolysaccharide synthesis | mru_1066 | mru_1066 | glycosyl transferase GT4 family | -1.04 | 8.06 |
| CELLULAR PROCESSES |  |  |  |  |  |
| Stress response | mru_0183 | mru_0183 | protein disulfide-isomerase thioredoxin-related protein | 1.29 | 7.87 |
| CENTRAL CARBON METABOLISM | | |  |  |  |
| Other | mru_1685 | deoC | deoxyribose-phosphate aldolase DeoC | -1.53 | 8.02 |
| ENERGY METABOLISM | |  |  |  |  |
| H2 metabolism | mru_2064 | frhA | coenzyme F420 hydrogenase alpha subunit FrhA | 1.54 | 8.48 |
|  | mru_2061 | frhB1 | coenzyme F420 hydrogenase beta subunit FrhB1 | 1.31 | 7.91 |
|  | mru_2063 | frhD | coenzyme F420 hydrogenase delta subunit FrhD | 1.27 | 6.91 |
|  | mru_2062 | frhG | coenzyme F420 hydrogenase gamma subunit FrhG | 1.26 | 8.01 |
|  | mru_1632 | hypB | hydrogenase accessory protein HypB | -1.04 | 7.11 |
|  | mru_1633 | hypA | hydrogenase nickel insertion protein HypA | -1.16 | 6.80 |
| Electron transfer | mru_0184 | dsbD | cytochrome C-type biogenesis protein DsbD | 1.26 | 6.25 |
| Alcohol metabolism | mru_1445 | adh3 | NADP-dependent alcohol dehydrogenase Adh3 | -1.83 | 3.67 |
| Methanogenesis pathway | mru_0441 | mtrA2 | tetrahydromethanopterin S-methyltransferase subunit A MtrA2 | 2.93 | 11.98 |
| LIPID METABOLISM | |  |  |  |  |
| Biosynthesis bacterial | mru_1630 | fabG2 | 3-oxoacyl-(acyl-carrier-protein) reductase FabG2 | 1.24 | 8.30 |
| MOBILE ELEMENTS | |  |  |  |  |
| Prophage | mru_0269 | mru_0269 | ATPase involved in DNA replication control MCM family | -2.28 | 4.52 |
|  | mru_0259 | mru_0259 | cdc6 family replication initiation protein Cdc6-3 | -1.12 | 5.86 |
|  | mru_0280 | mru_0280 | ParB-like nuclease domain-containing protein | -2.64 | 2.11 |
|  | mru_0256 | mru_0256 | phage integrase | -2.52 | 7.74 |
|  | mru_0287 | mru_0287 | phage portal protein | -2.47 | 1.78 |
|  | mru_0315 | mru_0315 | phage tail tape measure protein | -2.43 | 3.48 |
|  | mru_0307 | mru_0307 | phage-related protein | -3.27 | 2.90 |
|  | mru_0308 | mru_0308 | phage-related protein | -3.18 | 3.31 |
|  | mru_0313 | mru_0313 | phage-related protein | -2.96 | 2.57 |
|  | mru_0311 | mru_0311 | phage-related protein | -2.85 | 2.43 |
|  | mru_0310 | mru_0310 | phage-related protein | -2.84 | 1.40 |
|  | mru_0284 | mru_0284 | phage-related protein | -2.76 | 1.56 |
|  | mru_0317 | mru_0317 | phage-related protein | -2.65 | 3.34 |
|  | mru_0282 | mru_0282 | phage-related protein | -2.49 | 1.94 |
|  | mru_0316 | mru_0316 | phage-related protein | -2.36 | 3.27 |
|  | mru_0288 | mru_0288 | phage-related protein | -2.35 | 2.56 |
|  | mru_0270 | mru_0270 | phage-related protein | -1.62 | 4.43 |
|  | mru_0285 | mru_0285 | terminase large subunit | -1.05 | 4.24 |
| NITROGEN METABOLISM | |  |  |  |  |
| Other | mru_2121 | hcp | hydroxylamine reductase Hcp | 2.35 | 12.19 |
| PROTEIN FATE |  |  |  |  |  |
| Protein degradation | mru_1028 | mru_1028 | peptidase C39 family | 1.09 | 1.62 |
| PROTEIN SYNTHESIS | |  |  |  |  |
| RNA processing | mru_0589 | mru_0589 | NMD3 family protein | -1.02 | 7.29 |
| Ribosomal proteins | mru_0862 | mru_0862 | ribosomal protein L14P Rpl14p | -1.09 | 8.16 |
|  | mru_0856 | mru_0856 | ribosomal protein L22P Rpl22p | -1.01 | 8.22 |
|  | mru_0863 | mru_0863 | ribosomal protein L24P Rpl24p | -1.04 | 7.29 |
|  | mru_0858 | mru_0858 | ribosomal protein L29P Rpl29p | -1.08 | 7.23 |
|  | mru_0851 | rpl3p | ribosomal protein L3P Rpl3p | -1.03 | 9.38 |
|  | mru_0865 | rpl5p | ribosomal protein L5P Rpl5p | -1.05 | 8.37 |
|  | mru_0864 | rps4e | ribosomal protein S4e Rps4e | -1.01 | 9.03 |
| Translation factors | mru_0859 | mru_0859 | translation initiation factor aSUI1 | -1.03 | 7.32 |
| REGULATION |  |  |  |  |  |
| Transcriptional regulator | mru_0430 | mru_0430 | transcriptional regulator | 1.11 | 2.63 |
|  | mru_2122 | mru_2122 | transcriptional regulator | 2.47 | 8.63 |
|  | mru_1334 | mru_1334 | transcriptional regulator ArsR family | 2.32 | 2.65 |
|  | mru_0132 | mru_0132 | transcriptional regulator ArsR family | 2.61 | 8.34 |
|  | mru_0442 | mru_0442 | transcriptional regulator MarR family | 1.04 | 5.03 |
| TRANSPORTERS |  |  |  |  |  |
| Amino acids | mru_1775 | mru_1775 | amino acid ABC transporter ATP-binding protein | -1.26 | 5.73 |
|  | mru_1776 | mru_1776 | amino acid ABC transporter permease protein | -1.01 | 4.89 |
| Cations | mru_0537 | feoB2 | ferrous iron transport protein B FeoB2 | 1.33 | 5.00 |
|  | mru_1861 | mru_1861 | heavy metal translocating P-type ATPase | 5.53 | 10.37 |
|  | mru_1333 | mru_1333 | heavy metal-translocating P-type ATPase | 2.34 | 6.67 |
|  | mru_1706 | nikD2 | nickel ABC transporter ATP-binding protein NikD2 | -1.05 | 6.59 |
|  | mru_0207 | mru_0207 | potassium uptake protein TrkH family | 1.47 | 6.70 |
| Other | mru_0253 | mru_0253 | ABC transporter ATP-binding protein | 1.13 | 5.60 |
|  | mru_0252 | mru_0252 | ABC transporter permease protein | 1.23 | 5.94 |
|  | mru_0251 | mru_0251 | ABC transporter substrate-binding protein | 1.15 | 7.41 |
| VITAMINS AND COFACTORS | |  |  |  |  |
| Ubiquinone | mru_1969 | ubiB4 | 2-polyprenylphenol 6- hydroxylase UbiB4 | 1.10 | 10.19 |
| Riboflavin | mru_0089 | ribB | 3,4-dihydroxy-2-butanone 4-phosphate synthase RibB | -1.07 | 8.65 |
| Coenzyme B | mru_0385 | aksA | homocitrate synthase AksA | 1.34 | 10.27 |
| Nicotinate | mru_1750 | mru_1750 | nicotinate phosphoribosyltransferase | -1.30 | 9.78 |
| Others | mru_0734 | mru_0734 | 5-formyltetrahydrofolate cyclo-ligase | -1.09 | 5.23 |
| Others | mru_1769 | nifB | nitrogenase cofactor biosynthesis protein NifB | -1.09 | 7.84 |
| UNKNOWN FUNCTION | |  |  |  |  |
| Enzyme | mru_2170 | mru_2170 | acetyltransferase | -1.02 | 6.03 |
|  | mru_1758 | mru_1758 | acetyltransferase | 1.18 | 6.13 |
|  | mru_0574 | mru_0574 | acetyltransferase GNAT family | 1.37 | 2.02 |
|  | mru_1026 | mru_1026 | SAM-dependent methyltransferase | 1.59 | 6.11 |
| Other | mru_1510 | mru_1510 | YhgE/Pip-like protein | 1.08 | 8.63 |
|  | mru_0627 | mru_0627 | ZPR1 zinc-finger domain-containing protein | -1.06 | 6.08 |
