# Supplementary material for: The transcriptome response of the ruminal methanogen Methanobrevibacter ruminantium strain M1 to the inhibitor lauric acid
Source: BMC Res Notes. 2018 Feb 17;11:135. doi: 10.1186/s13104-018-3242-8 (PMC5816558; doi:10.1186/s13104-018-3242-8)
Supplement: Supplementary file 2 — Additional file 2: Table S2. M. ruminantium M1 genes with significantly changed expression of genes in the cultures exposed to C12 + DMSO as compared to the blank group (log2-fold change < 1 and > 1, false discovery rate < 0.05). The list does not include the 15 regulated hypothetical proteins. The M. ruminantium (mru) open reading frame (ORF) codes are adopted from the Kyoto Encyclopedia of Genes and Genomes. [file 13104_2018_3242_MOESM2_ESM.docx]

**Additional file 2: Table S2. *M. ruminantium* M1 genes with significantly changed expression of genes in the cultures exposed to C_12_ +DMSO as compared to the blank group (log_2_-fold change < 1 and > 1, false discovery rate < 0.05).** The list does not include the 15 regulated hypothetical proteins. The *M. ruminantium* (mru) open reading frame (ORF) codes are adopted from the Kyoto Encyclopedia of Genes and Genomes.

| **Category and Subcategory** | **ORF** | **Gene name** | **Annotated function** | **log2-fold change** | **log2 counts per 10^6^ reads** |
| --- | --- | --- | --- | --- | --- |
| AMINO ACID METABOLISM | |  |  |  |  |
| Glutamate/ glutamine | mru_0350 | glnA1 | glutamine synthetase GlnA1 | 1,04 | 9,53 |
| Methionine | mru_1569 | mru_1569 | O-acetylhomoserine/O-acetylserine sulfhydrylase MetZ/CysK2 | -1,53 | 6,71 |
| Tryptophan | mru_0214 | trpA | tryptophan synthase alpha subunit TrpA | -1,12 | 8,77 |
| CELL ENVELOPE |  |  |  |  |  |
| Cell surface proteins | mru_0086 | mru_0086 | adhesin-like protein | -1,49 | 10,76 |
| Cell surface proteins | mru_0076 | mru_0076 | adhesin-like protein | -1,37 | 10,76 |
| Cell surface proteins | mru_0727 | mru_0727 | adhesin-like protein with cysteine protease domain | -1,10 | 9,15 |
| Cell surface proteins | mru_0327 | mru_0327 | adhesin-like protein | 2,13 | 9,91 |
| Cell surface proteins | mru_0326 | mru_0326 | adhesin-like protein | 3,74 | 10,83 |
| CELLULAR PROCESSES | |  |  |  |  |
| Oxidative stress response | mru_1367 | rbr2 | rubrerythrin Rbr2 | 1,65 | 13,25 |
| Oxidative stress response | mru_0131 | fprA2 | F420H2 oxidase FprA2 | 2,15 | 11,74 |
| CENTRAL CARBON METABOLISM | | |  |  |  |
| Butanol | mru_0990 | hbd | 3-hydroxybutyryl-CoA dehydrogenase Hbd | 1,33 | 2,91 |
| Other | mru_1685 | deoC | deoxyribose-phosphate aldolase DeoC | 2,87 | 10,61 |
| ENERGY METABOLISM | | |  |  |  |
| Alcohol metabolism | mru_1444 | npdG2 | NADPH-dependent F420 reductase NpdG2 | 2,41 | 4,07 |
| H2 metabolism | mru_2062 | frhG | coenzyme F420 hydrogenase gamma subunit FrhG | 1,24 | 7,97 |
| H2 metabolism | mru_2063 | frhD | coenzyme F420 hydrogenase delta subunit FrhD | 1,36 | 6,94 |
| H2 metabolism | mru_2061 | frhB1 | coenzyme F420 hydrogenase beta subunit FrhB1 | 1,38 | 7,93 |
| H2 metabolism | mru_2064 | frhA | coenzyme F420 hydrogenase alpha subunit FrhA | 1,51 | 8,43 |
| Methanogenesis pathway | mru_0441 | mtrA2 | tetrahydromethanopterin S-methyltransferase subunit A MtrA2 | 3,14 | 12,14 |
| MOBILE ELEMENTS |  |  |  |  |  |
| Prophage | mru_0273 | mru_0273 | hypothetical protein | -1,95 | 1,61 |
| Prophage | mru_0256 | mru_0256 | phage integrase | -1,74 | 7,85 |
| Prophage | mru_0259 | mru_0259 | cdc6 family replication initiation protein Cdc6-3 | -1,19 | 5,81 |
| NITROGEN METABOLISM | | | |  |  |
| Other | mru_2121 | hcp | hydroxylamine reductase Hcp | 2,22 | 12,05 |
| PROTEIN FATE |  |  |  |  |  |
| Protein degradation | mru_1028 | mru_1028 | peptidase C39 family | 1,86 | 2,12 |
| REGULATION |  |  |  |  |  |
| Transcriptional regulator | mru_2122 | mru_2122 | transcriptional regulator | 1,92 | 8,14 |
| Transcriptional regulator | mru_1334 | mru_1334 | transcriptional regulator ArsR family | 2,17 | 2,47 |
| TRANSPORTERS |  |  |  |  |  |
| Amino acids | mru_1775 | mru_1775 | amino acid ABC transporter ATP-binding protein | -1,03 | 5,77 |
| Cations | mru_0537 | feoB2 | ferrous iron transport protein B FeoB2 | 1,49 | 5,08 |
| Cations | mru_1333 | mru_1333 | heavy metal-translocating P-type ATPase | 2,09 | 6,43 |
| Cations | mru_1861 | mru_1861 | heavy metal translocating P-type ATPase | 3,57 | 8,46 |
| Other | mru_0329 | mru_0329 | MotA/TolQ/ExbB proton channel family protein | 1,32 | 5,27 |
| Other | mru_0253 | mru_0253 | ABC transporter ATP-binding protein | 1,84 | 6,08 |
| Other | mru_0252 | mru_0252 | ABC transporter permease protein | 1,92 | 6,42 |
| Other | mru_0251 | mru_0251 | ABC transporter substrate-binding protein | 1,96 | 7,98 |
| VITAMINS AND COFACTORS | |  |  |  |  |
| Metal-binding pterin | mru_0200 | modB | molybdate ABC transporter permease protein ModB | 1,54 | 8,58 |
| Metal-binding pterin | mru_0201 | modA | molybdate ABC transporter substrate-binding protein ModA | 2,42 | 9,58 |
| Methanopterin | mru_1690 | mptG | beta-ribofuranosylaminobenzene 5'-phosphate synthase MptG | 1,10 | 6,80 |
| Nicotinate | mru_1750 | mru_1750 | nicotinate phosphoribosyltransferase | -1,35 | 9,73 |
| Others | mru_1769 | nifB | nitrogenase cofactor biosynthesis protein NifB | 1,46 | 9,16 |
| Ubiquinone | mru_1969 | ubiB4 | 2-polyprenylphenol 6- hydroxylase UbiB4 | 1,14 | 10,18 |
| UNKNOWN FUNCTION | |  |  |  |  |
| Enzyme | mru_1854 | mru_1854 | ATPase | 1,01 | 9,14 |
| Enzyme | mru_1026 | mru_1026 | SAM-dependent methyltransferase | 1,58 | 6,06 |
| Other | mru_1034 | mru_1034 | HEAT repeat-containing protein | 1,06 | 8,54 |
