# Supplementary material for: The transcriptome response of the ruminal methanogen Methanobrevibacter ruminantium strain M1 to the inhibitor lauric acid
Source: BMC Res Notes. 2018 Feb 17;11:135. doi: 10.1186/s13104-018-3242-8 (PMC5816558; doi:10.1186/s13104-018-3242-8)

**Additional file 3: Figure S1.** **Venn diagram indicates the number of differentially expressed genes between the experimental groups and the common overlapping differentially expressed genes.** TC: treatment (C_12_+DMSO) vs. control (DMSO); TB: treatment (C_12_+DMSO) vs. untreated blank; CB: control (DMSO) vs. untreated blank. It should be kept in mind that it is not possible to distinguish between the DMSO and the C_12_ effect in the dataset comparing the treatment and the blank samples, and that the C_12_ effect is much better studied in the TC comparison (C_12_+DMSO vs DMSO). The DMSO effect can be partial quenched by the C_12_ effect, so genes regulated in CB and TC are not necessarily regulated in the TB. The 26 common genes differentially expressed in *M. ruminantium* exposed to DMSO or DMSO+C_12_ compared to the untreated blank control are outlined in the tables on the right side. The 35 overlapping differentially expressed genes of the TC and CB comparisons are outlined in the table on the left side. The diagram was generated using the online tool at bioinformatics.psb.ugent.be/webtools/Venn/.


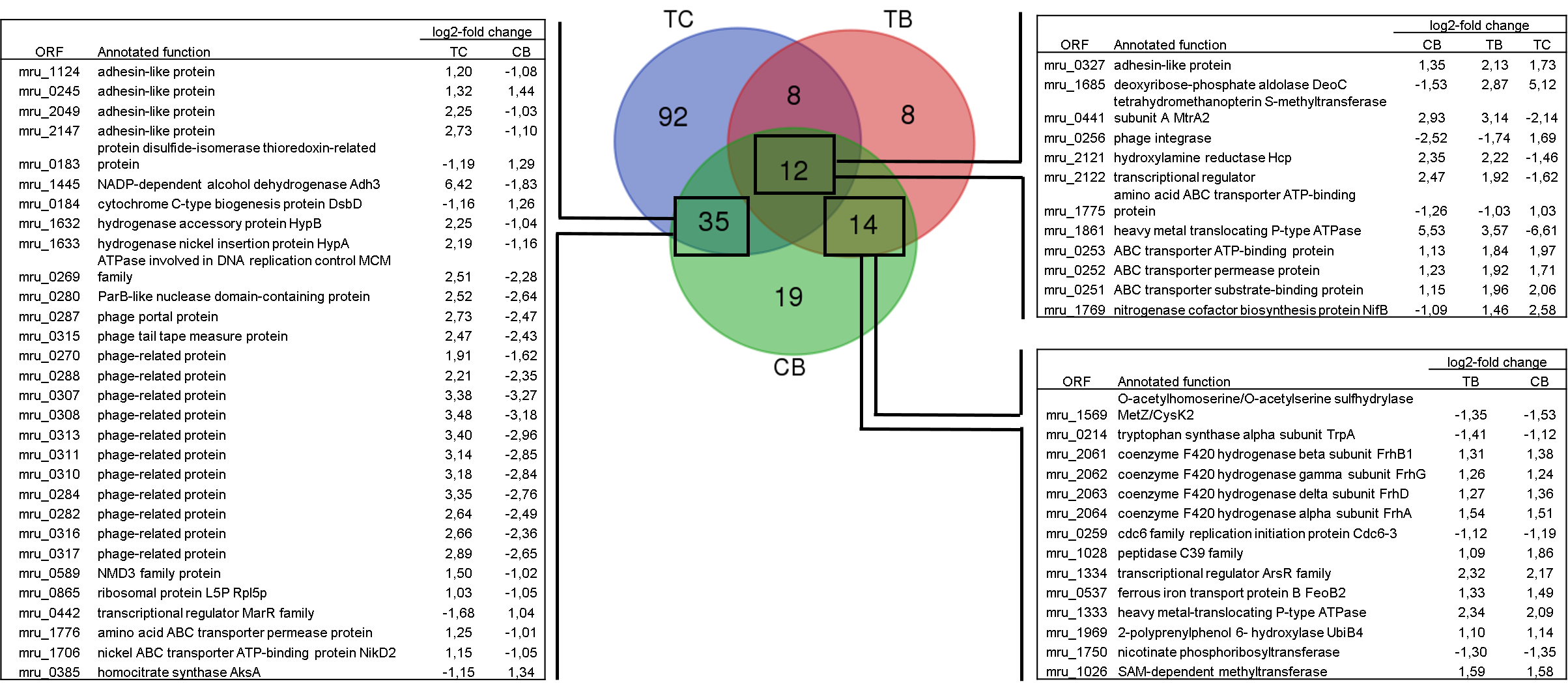

Supplement: Supplementary file 3 — Additional file 3: Figure S1. Venn diagram indicates the number of differentially expressed genes between the experimental groups and the common overlapping differentially expressed genes. TC: treatment (C12 + DMSO) vs. control (DMSO); TB: treatment (C12 + DMSO) vs. untreated blank; CB: control (DMSO) vs. untreated blank. It should be kept in mind that it is not possible to distinguish between the DMSO and the C12 effect in the dataset comparing the treatment and the blank samples, and that the C12 effect is much better studied in the TC comparison (C12 + DMSO vs DMSO). The DMSO effect can be partial quenched by the C12 effect, so genes regulated in CB and TC are not necessarily regulated in the TB. The 26 common genes differentially expressed in M. ruminantium exposed to DMSO or DMSO + C12 compared to the untreated blank control are outlined in the tables on the right side. The 35 overlapping differentially expressed genes of the TC and CB comparisons are outlined in the table on the left side. The diagram was generated using the online tool at bioinformatics.psb.ugent.be/webtools/Venn/. [file 13104_2018_3242_MOESM3_ESM.docx]
